# Artificial intelligence-based thigh muscle volume profiling on CT in elderly East Asian patients with hip fracture

**DOI:** 10.1371/journal.pone.0359317

**Published:** 2026-10-08

**Authors:** Yong-Han Cha, Jung-Taek Kim, Jin-Woo Kim, Hyun-Bin Kim, Jun-Il Yoo

**Affiliations:** 1 Department of Orthopaedic Surgery, Daejeon Eulji Medical Center, Eulji University School of Medicine, Daejeon, Republic of Korea; 2 Department of Orthopaedic Surgery, Ajou University School of Medicine, Ajou Medical Center, Suwon, Republic of Korea; 3 Department of Orthopaedic Surgery, Nowon Eulji Medical Center, Eulji University, Seoul, Republic of Korea; 4 Program in Biomedical Science and Engineering, Inha University, Incheon, Republic of Korea; 5 Department of Orthopaedic Surgery, Inha University Hospital, Inha University College of Medicine, Incheon, Republic of Korea; Shuguang Hospital Affiliated to Shanghai University of Traditional Chinese Medicine, CHINA

## Abstract

**Background:**

Skeletal muscle mass and quality are critical determinants of outcomes in hip fracture patients. However, most prior studies have relied on 2D cross-sectional area measurements, which may not accurately reflect the complex 3D muscle distribution. This study aimed to quantify 3D thigh muscle volume and intramuscular adipose tissue (IMAT) in a large elderly East Asian hip fracture cohort using an AI-assisted segmentation pipeline.

**Methods:**

A total of 1,287 patients from a multi-center hip fracture cohort were included. Preoperative whole-thigh CT scans were analyzed using a validated AI-based 3D UNETR segmentation model to delineate 26 individual muscles. Height-normalized muscle volumes (cm³/m²) and IMAT percentages (%) were computed for four anatomic regions: anterior, medial, posterior, and gluteal.

**Results:**

The gluteal group exhibited the largest height-normalized muscle volume (855.64 ± 216.38 cm³/m²), followed by the anterior group (784.57 ± 243.17 cm³/m²). On an individual level, the gluteus maximus was the largest single muscle. Paradoxically, the gluteal group showed the highest IMAT percentage (1.43 ± 0.84%), higher than the anterior (0.57 ± 0.42%) and medial groups (0.71 ± 0.51%). Specifically, the highest IMAT percentages were observed in the multifidus (1.93 ± 1.31%) and tensor fasciae latae (1.92 ± 1.03%), which contrasts with the relatively preserved primary locomotive muscles such as the vastus medialis (0.38 ± 0.32%).

**Conclusions:**

Elderly hip fracture patients exhibited a dissociation between muscle quantity and quality. The relatively high fatty degeneration observed in stabilizing muscles, including the gluteal muscles and multifidus, despite their large volume, may reflect muscle composition patterns potentially related to postural function. These findings should be considered hypothesis-generating and require validation in prospective studies incorporating direct functional assessments.

## Introduction

Hip fractures are among the most devastating injuries in older adults, often leading to prolonged immobility, functional decline, institutionalization, and increased mortality [1]. Despite advances in surgical techniques and postoperative care, up to one-third of patients die within one year after fracture, and many survivors fail to regain their pre-fracture level of independence [2]. These poor outcomes are strongly influenced by frailty and sarcopenia the age-related loss of skeletal muscle mass and function [3,4]. Previous studies have demonstrated that patients with hip fractures exhibit marked reductions in thigh muscle volume and strength compared with age-matched controls, and that low muscle mass is independently associated with higher rates of postoperative complications, delayed ambulation, and readmission [5–7]. Therefore, accurate assessment of skeletal muscle quantity and quality is important for characterizing musculoskeletal status in hip fracture patients and may help inform targeted rehabilitation strategies [8].

Quantitative evaluation of muscle volume provides a direct indicator of musculoskeletal integrity and functional reserve [5]. Imaging modalities including magnetic resonance imaging (MRI), computed tomography (CT), and dual-energy X-ray absorptiometry (DXA) have been used to assess muscle size or volume [9,10]. Among them, MRI and CT allow 3-dimensional reconstruction of muscle volume and visualization of fat infiltration, which are critical markers of muscle quality [11,12]. Several MRI-based studies have established normative data for thigh muscle compartments and documented age-related atrophy, with the quadriceps especially the rectus femoris showing the greatest decline in older adults [13,14]. However, most prior investigations analyzed only a few selected cross-sectional slices and extrapolated total muscle volume, rather than computing it across the entire thigh. Furthermore, these studies were largely confined to small numbers of participants, even when numerous, typically included only healthy volunteers. Thus, their results are difficult to extrapolate to acutely ill or highly frail populations like patients suffering from hip fractures [3,7]. To overcome these limitations, a comprehensive volumetric analysis based on clinical imaging is needed. In the present study, we utilized preoperative CT scans that extended from the iliac wing to below the knee joint in a large hip fracture cohort. From each axial slice, the cross-sectional areas of individual muscles were measured using an artificial intelligence (AI)-assisted segmentation algorithm trained to recognize anatomical muscle boundaries [15,16]. By integrating all slices along the femoral axis, we computed the true three-dimensional volume of each muscle, allowing precise quantification of muscle loss in this high-risk population.

Therefore, the objective of this study was to quantify CT-derived thigh muscle volume in elderly East Asian patients with hip fracture, to characterize its distribution and clinical features in both muscle volume and intramuscular fat content.

## Materials and methods

### Study population

This study utilized CT images from patients enrolled in a multi-center hip fracture cohort, a hybrid registry comprising both retrospectively collected and prospectively enrolled data from elderly patients with fragility hip fractures [17]. Cohort was designed to integrate clinical, imaging, and functional information across participating institutions. Patients aged over 50 years who sustained a fragility-related hip fracture were included in the registry. The present study included clinical and preoperative whole-thigh CT imaging data acquired between October 2015 and July 2024 from patients treated at five university-affiliated tertiary hospitals. Demographic and clinical data were collected through a secure digital registry. The authors accessed the de-identified clinical and imaging data for research purposes between 01/01/2025 and 28/02/2025. For the present analysis, we included patients who met the following criteria: hip fracture resulting from a low-energy trauma mechanism; femoral neck or intertrochanteric fracture; availability of preoperative whole-thigh CT imaging; and complete, accessible medical records. Patients were excluded if they had a pathologic fracture, a subtrochanteric or atypical fracture, a history of surgery on the contralateral femur or pelvis, or if the presence of implants made muscle volume assessment on CT infeasible (Fig 1).

**Fig 1:**
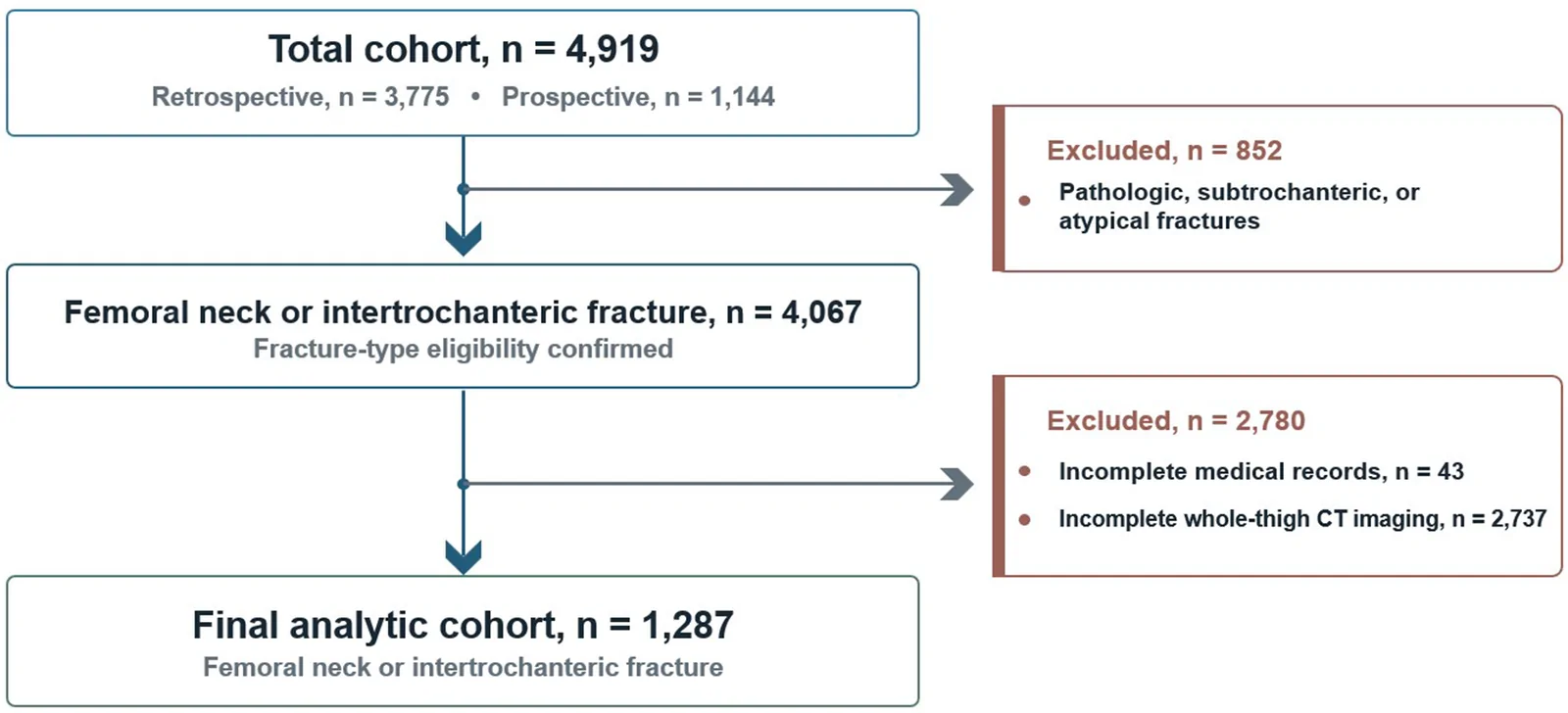
Research flow diagram of patient inclusion.

This multicenter study was approved by the Institutional Review Board of Inha University Hospital, the coordinating institution for the study (IRB No. 2023-04-027), and this approval covered the participating institutions. The study was conducted in accordance with the Declaration of Helsinki and applicable institutional guidelines. The requirement for informed consent was waived due to the retrospective analysis of de-identified clinical and imaging data.

### CT acquisition

All whole-thigh CT scans were performed with the patient in the supine position (head-first or feet-first) and covered the entire region from the hip to the knee joint [18]. The scanning range typically extended from the L3–L4 vertebral level to the distal femur to ensure complete visualization of the thigh musculature. CT images with metallic artifacts, incomplete thigh coverage, severe deformities were excluded to maintain segmentation quality [18,19]. Because CT examinations were retrospectively collected across participating institutions, acquisition and reconstruction parameters were not completely uniform across all scans. Available source DICOM metadata demonstrated variability in scanner platform, tube voltage, in-plane pixel spacing, matrix dimensions, and reconstruction kernel. Therefore, acquisition heterogeneity was considered an inherent feature of the multicenter imaging dataset.

### Image preprocessing

To enhance muscle–fat boundary visibility, voxel intensity was rescaled from −57 to +164 Hounsfield units, and gamma correction (γ = 2.0) was applied to improve local contrast and minimize artifact effects [18,19]. Images were resampled to isotropic resolution and cropped to include only the thigh region prior to segmentation. The same preprocessing procedure was applied to all images before segmentation to reduce input variability across scans and was consistent with the preprocessing approach used in the previous model-validation work.

### AI-based muscle segmentation pipeline

Quantitative analysis of muscle volume was conducted using a previously developed and validated deep-learning segmentation pipeline. The segmentation pipeline was developed and evaluated in a previously published study using 72 manually annotated whole-thigh CT scans from patients with hip fracture. The 3D UNETR (U-Net Transformer) model was trained and validated using this annotated dataset and achieved a mean Dice similarity coefficient of 0.84 and a relative average volume difference of 0.019%. These performance metrics were derived from the previous model development and validation study and were not obtained from a new validation analysis of the present 1,287-patient cohort. In the current study, the previously developed model was applied to the multicenter hip-fracture cohort to automatically segment individual muscles and quantify muscle volume and adipose tissue distribution [18,19].

The segmentation model was used to delineate 26 individual muscles, which were categorized into four primary anatomic regions (anterior, medial, posterior, and gluteal) and an additional “other” compartment. Because the CT scan initiation level varied between L3 and L4, truncal muscles that were not consistently visualized were excluded from detailed analysis (Fig 2). The anterior group comprised the sartorius, rectus femoris, and the vastus muscles, including the vastus lateralis, vastus intermedius, and vastus medialis. The medial group consisted of the adductor magnus, adductor brevis, adductor longus, gracilis, and pectineus. The posterior group included the biceps femoris, semitendinosus, and semimembranosus. The gluteal group encompassed the gluteus maximus, gluteus medius, gluteus minimus, tensor fasciae latae, piriformis, quadratus femoris, obturator internus, and obturator externus. Finally, other truncal and hip structures, including the iliacus, iliopsoas, abdominal oblique, rectus abdominis, and multifidus, were grouped into a separate compartment. In total, 21 muscles were included in the four primary anatomical compartments (5 anterior, 5 medial, 3 posterior, and 8 gluteal), while 5 additional muscles or structures were classified in the “other” compartment, yielding 26 muscles in the present analysis. Primary anatomical-compartment comparisons and the corresponding individual-muscle inferential analyses were restricted to the 21 muscles comprising the four primary compartments. Measurements from the additional “other” compartment were retained for descriptive reporting in Table 2 but were not included in these primary inferential comparisons.

**Fig 2:**
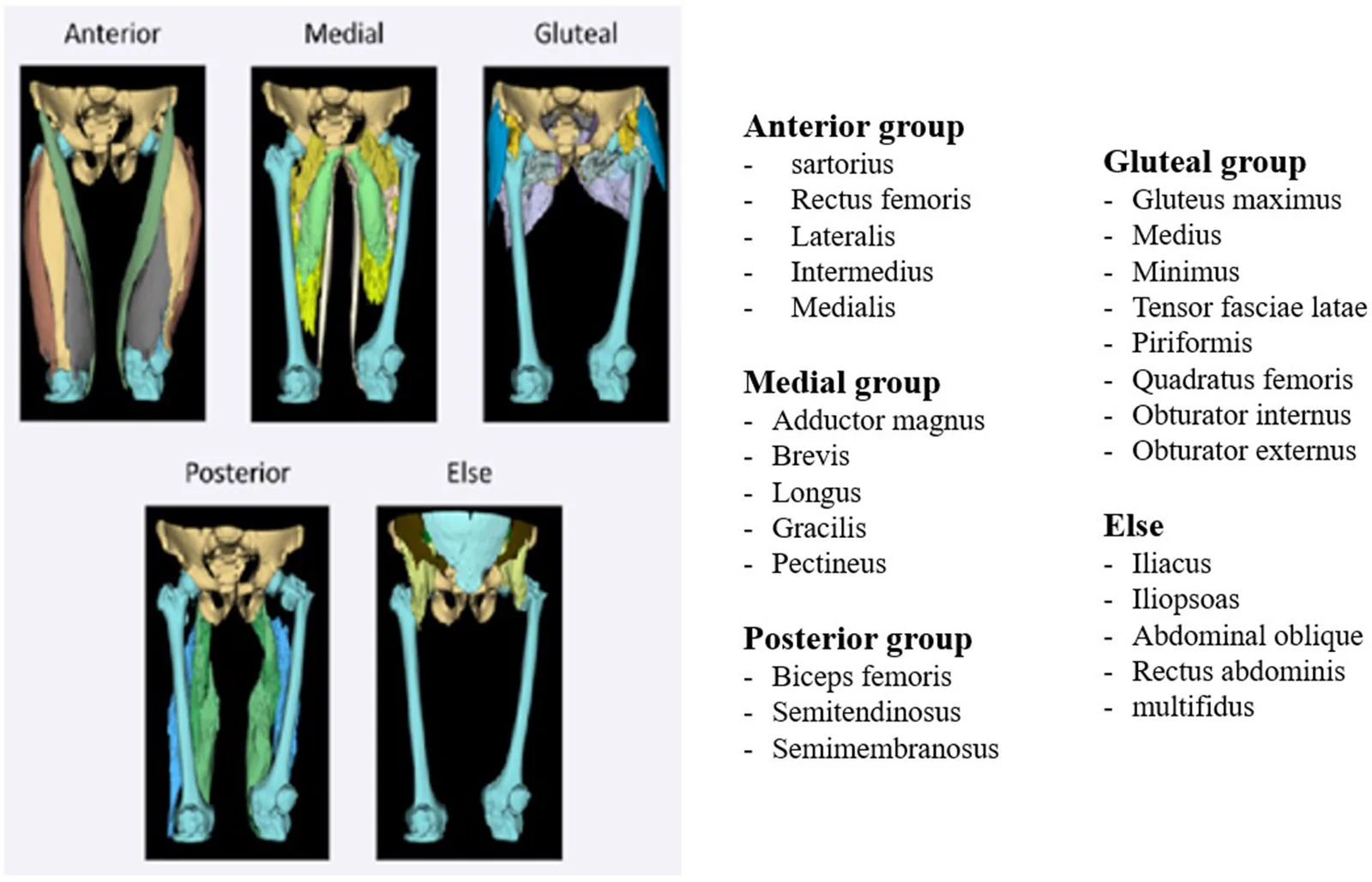
Representative 3D renderings of AI-based segmentation of individual thigh muscle groups.

### Muscle volume and IMAT calculation

Muscle volume (cm³) was computed as the sum of voxel volumes (pixel width × pixel height × slice thickness) within each labeled muscle region. Volumes were normalized by height squared (cm³/m²) to account for body size variation. Height-normalized muscle volume (cm³/m²) was used consistently for descriptive summaries, anatomical-compartment comparisons, age-related analyses, and multivariable regression. Absolute volume in cm³ was retained only as an intermediate quantity used to derive the normalized measure. Intramuscular adipose tissue (IMAT) was operationally defined as voxels with attenuation values between −190 and −30 HU located within each individual muscle segmentation mask on the original, non-normalized CT image. After inference, the predicted muscle maps were transformed back to the original CT physical coordinate system, including reversal of test-time augmentation when used, padding, and cropping. For each individual muscle, IMAT voxels were defined as the intersection between the corresponding muscle segmentation mask and voxels within the −190 to −30 HU range. Adipose-range voxels outside the corresponding muscle mask were therefore excluded directly by the anatomical muscle mask. No fat-specific morphological filtering, hole filling, or boundary smoothing was applied. Absolute IMAT volume was calculated by summing the physical voxel volumes of all voxels within each muscle mask that satisfied the −190 to −30 HU criterion, based on the original CT spatial information. IMAT percentage was calculated as the IMAT volume within the muscle mask divided by the total segmented muscle volume and multiplied by 100.

### Data integration and statistical analysis

Continuous variables were expressed as mean ± standard deviation (SD), and categorical variables were summarized as frequencies and percentages. Between-group comparisons for categorical variables were performed using chi-square or Fisher’s exact tests, and continuous variables were compared using independent t-tests. For age-related analyses, patients were stratified into three predefined age categories (< 65 years, 65–84 years, and ≥ 85 years). Differences in muscle and IMAT volumes across the three age groups were evaluated using one-way analysis of variance (ANOVA), followed by appropriate post hoc tests when overall significance was observed.

For comparisons among anatomical compartments, repeated-measures ANOVA was used because measurements from multiple compartments were obtained within each patient. Greenhouse–Geisser-corrected results were additionally evaluated, and post hoc pairwise comparisons were performed using two-sided paired t-tests with Holm–Bonferroni correction across the six compartment comparisons. Individual-muscle comparisons among the 21 muscles comprising the four primary anatomical compartments were considered a separate exploratory family, with Holm–Bonferroni correction applied across all 210 pairwise comparisons. An additional sensitivity analysis applied the Holm–Bonferroni correction across all 216 compartment- and muscle-level comparisons combined. To further examine sex- and age-related differences while accounting for potential confounders, multiple linear regression analyses were performed.

Because muscle and IMAT volumes were normalized to height squared (cm³/m²), height and weight were not entered separately as covariates. BMI was retained as an adjustment variable to account for differences in overall body habitus and adiposity beyond height normalization. Multicollinearity among model covariates was assessed using variance inflation factors (VIFs). Sensitivity analyses were additionally performed by comparing otherwise identical multivariable models with and without BMI. Models were additionally adjusted for age group, sex, fracture side, fracture type, pre-injury Koval grade, Functional Independence Measure (FIM), Charlson Comorbidity Score, anesthesia type, and surgery type [20–22]. Regression coefficients (β) and 95% confidence intervals (CIs) were reported. Statistical significance was defined as p < 0.05.

To quantify the associations of age, sex, and anatomical muscle group with muscle volume and IMAT while accounting for repeated muscle-group measurements within individuals, additional linear mixed-effects models were fitted using the data in long format. Patient-specific random intercepts were included, with age, sex, muscle group, and BMI entered as fixed effects. Female sex and the anterior muscle group were used as reference categories. Height-normalized muscle volume (cm³/m²) and IMAT percentage were analyzed as separate outcomes.

## Results

A total of 1,287 hip fracture patients were included in this study, with a mean age of 81.1 ± 10.1 years and a female predominance (66.8%). Fracture types were distributed between intertrochanteric (53.8%) and femoral neck fractures (46.2%), with balanced laterality (52.2% left vs. 47.8% right). Regarding baseline functional status, 52% of the cohort were classified as Koval grade 1, indicating pre-injury functional independence. The mean Charlson Comorbidity Score was 4.8 ± 1.2. Surgical management was nearly equally divided between internal fixation (53.4%) and arthroplasty (46.6%) (Table 1).

**Table 1:** Baseline characteristics of included hip fracture patients.

Variables	Total (n = 1,287)
Age (years, mean±SD)	81.1 ± 10.1
Sex (men/women, n(%))	427(33.2%)/860(66.8%)
Height (cm, mean±SD)	158.8 ± 9.0
Weight (kg, mean±SD)	56.0 ± 10.6
Body mass index (kg/m², mean±SD)	22.2 ± 3.6
Fracture side (left/right, n (%))	672(52.2%)/615(47.8%)
Fracture type (intertrochanter/neck, n (%))	693(53.8%)/594(46.2%)
Pre-injury Koval grade (1/2/3/4/5/6/7, n (%))	672(52%)/200(16%)/89(7%)/137(11%)/69(5%)/81(6%)/39(3%)
Functional Independence Measure (Complete Independence/Modified Independence/Supervision/Minimal Assistance/Moderate Assistance/Maximal Assistance/Total Assistance, n (%))	672(52%)/234(18%)/72(6%)/124(10%)/48(4%)/81(6%)/56(4%)
Charlson Comorbidity Score (mean±SD)	4.8 ± 1.2
Anesthesia (general/spinal/epidural, n(%))	670(52%)/611(47.5%)/6(0.5%)
Surgery type (arthroplasty/internal fixation, n (%))	600(46.6%)/687(53.4%)

SD, standard deviation; n, number.

Comparative analysis by sex revealed that women were significantly older than men (82.5 ± 9.3 years vs. 78.3 ± 11.0 years, p < 0.001). While men were significantly taller (167.9 cm vs. 154.2 cm, p < 0.001) and heavier (61.7 kg vs. 53.2 kg, p < 0.001) than women, there was no significant difference in Body Mass Index (BMI) between the two groups (p = 0.318). Pre-injury functional status also differed by sex, with a significantly higher proportion of men being classified as Koval grade 1 (56.7%) compared to women (50.0%, p = 0.041). Additionally, a significant difference was observed in the type of anesthesia used (p = 0.033), with women undergoing general anesthesia at a slightly higher rate than men. However, no statistically significant differences were found between sexes regarding fracture side (p = 0.813), fracture type (p = 0.953), FIM scores (p = 0.087), Charlson Comorbidity Score (p = 0.059), or the type of surgery performed (p = 0.407).

The three-dimensional volumetric analysis of the thigh, derived from AI-based CT segmentation, provided detailed profiles of muscle and IMAT distribution (Table 2). Among the four anatomic groups, the gluteal group exhibited the largest height-normalized muscle volume (855.64 ± 216.38 cm³/m²), followed by the anterior group (784.57 ± 243.17 cm³/m²), medial group (514.44 ± 165.55 cm³/m²), and posterior group (308.31 ± 90.79 cm³/m²). On an individual muscle level, the gluteus maximus was the largest single muscle. The overall difference among the four anatomical compartments was significant on repeated-measures ANOVA (Greenhouse–Geisser-corrected F[2.60, 3339.83] = 7385.766, p < 0.001). Pairwise post hoc comparisons showed significant differences for all six anatomical-compartment pairs after Holm–Bonferroni correction: anterior versus medial (mean difference = 270.13 cm³/m², 95% CI 261.60 to 278.66), anterior versus posterior (476.27 cm³/m², 95% CI 466.83 to 485.71), anterior versus gluteal (−71.07 cm³/m², 95% CI −79.69 to −62.46), medial versus posterior (206.14 cm³/m², 95% CI 200.03 to 212.25), medial versus gluteal (−341.20 cm³/m², 95% CI −348.40 to −334.00), and posterior versus gluteal (−547.34 cm³/m², 95% CI −555.85 to −538.82), with all Holm-adjusted p-values < 0.001.

**Table 2:** Height-normalized muscle volume and IMAT composition in hip fracture patients (n = 1,287).

Muscles	Total muscle volume (cm³/m², mean±SD)	Pure muscle volume (cm³/m², mean±SD)	IMAT volume (cm³/m², mean±SD)	IMAT percentage (%, mean±SD)
**Anterior group**	**784.57 ± 243.17**	**746.79 ± 244.28**	**37.79 ± 21.66**	**0.57 ± 0.42**
Sartorius	62.95 ± 20.34	57.32 ± 19.96	5.63 ± 2.27	1.09 ± 0.57
Rectus femoris	96.78 ± 31.63	92.30 ± 31.60	4.48 ± 2.91	0.55 ± 0.43
Vastus lateralis	219.52 ± 75.04	207.69 ± 75.06	11.83 ± 7.42	0.65 ± 0.52
Vastus intermedius	223.76 ± 70.61	213.76 ± 70.89	10.00 ± 6.75	0.52 ± 0.42
Vastus medialis	181.56 ± 59.57	175.71 ± 59.87	5.85 ± 3.79	0.38 ± 0.32
**Medial group**	**514.44 ± 165.55**	**483.49 ± 166.49**	**30.96 ± 18.77**	**0.71 ± 0.51**
Adductor longus	65.19 ± 21.42	62.26 ± 21.42	2.94 ± 2.13	0.53 ± 0.46
Adductor brevis	64.08 ± 19.63	59.92 ± 19.86	4.16 ± 2.66	0.79 ± 0.60
Adductor magnus	308.37 ± 112.16	291.40 ± 112.32	16.97 ± 11.92	0.65 ± 0.52
Gracilis	42.61 ± 15.18	38.60 ± 14.84	4.01 ± 1.53	1.14 ± 0.53
Pectineus	34.19 ± 9.23	31.31 ± 9.24	2.88 ± 1.59	1.01 ± 0.64
**Posterior group**	**308.31 ± 90.79**	**285.45 ± 91.54**	**22.85 ± 12.34**	**0.90 ± 0.62**
Semitendinosus	84.53 ± 24.62	78.79 ± 24.43	5.74 ± 3.44	0.79 ± 0.54
Semimembranosus	96.06 ± 32.09	89.33 ± 32.56	6.73 ± 4.10	0.90 ± 0.73
Biceps femoris	127.72 ± 41.13	117.33 ± 41.26	10.39 ± 5.65	1.01 ± 0.73
**Gluteal group**	**855.64 ± 216.38**	**758.35 ± 223.97**	**97.30 ± 44.38**	**1.43 ± 0.84**
Gluteus maximus	447.32 ± 126.86	390.59 ± 129.41	56.73 ± 28.68	1.64 ± 1.06
Gluteus medius	188.15 ± 56.38	172.39 ± 57.27	15.76 ± 8.98	1.04 ± 0.73
Gluteus minimus	74.30 ± 19.66	66.77 ± 19.71	7.54 ± 3.56	1.24 ± 0.70
Tensor fascia latae	33.67 ± 11.61	28.83 ± 11.18	4.84 ± 2.08	1.92 ± 1.03
Piriformis	22.92 ± 6.62	20.20 ± 6.41	2.72 ± 1.24	1.47 ± 0.76
Obturator internus	27.00 ± 7.33	24.38 ± 7.40	2.62 ± 1.19	1.19 ± 0.66
Obturator externus	22.49 ± 9.37	20.07 ± 9.09	2.42 ± 1.28	1.35 ± 0.73
Quadratus femoris	39.79 ± 9.23	35.11 ± 9.17	4.67 ± 2.07	1.42 ± 0.70
**Others**	**169.31 ± 51.24**	**156.50 ± 49.98**	**12.80 ± 7.05**	**0.89 ± 0.56**
Iliacus	31.10 ± 10.82	28.97 ± 10.42	2.13 ± 1.43	0.78 ± 0.58
Iliopsoas	69.07 ± 23.22	65.56 ± 23.26	3.51 ± 2.57	0.61 ± 0.50
Abdominal oblique	17.13 ± 11.93	15.99 ± 11.24	1.13 ± 1.09	0.76 ± 0.61
Multifidus	32.69 ± 18.77	28.17 ± 17.01	4.51 ± 2.95	1.92 ± 1.32
Rectus abdominis	19.33 ± 13.20	17.81 ± 12.41	1.52 ± 1.29	0.93 ± 0.68

Values are mean ± SD. Total muscle volume, pure muscle volume, and IMAT volume are height-squared normalized values expressed as cm³/m². IMAT percentage was calculated for each patient and anatomical unit before summary statistics were generated.

In the linear mixed-effects analysis of height-normalized muscle volume, increasing age was associated with lower muscle volume (β = −2.05, 95% CI −2.85 to −1.24, p < 0.001), whereas male sex was associated with greater muscle volume (β = 117.95, 95% CI 100.49 to 135.41, p < 0.001). Compared with the anterior group, the medial (β = −270.13, 95% CI −278.27 to −261.99, p < 0.001) and posterior groups (β = −476.27, 95% CI −484.41 to −468.12, p < 0.001) had lower normalized muscle volumes, whereas the gluteal group had a higher normalized muscle volume (β = 71.07, 95% CI 62.93 to 79.21, p < 0.001). In the corresponding IMAT analysis, age was not significantly associated with IMAT percentage (β = 0.014, p = 0.215), whereas male sex was associated with a lower IMAT percentage (β = −2.22, p < 0.001). Relative to the anterior group, IMAT percentage was higher in the medial (β = 1.21, p < 0.001), posterior (β = 2.74, p < 0.001), and gluteal groups (β = 6.83, p < 0.001).

In terms of muscle quality, the gluteal group showed the highest IMAT percentage (1.43 ± 0.84%), which was notably higher than that of the anterior group (0.57 ± 0.42%) or the medial group (0.71 ± 0.51%). Among individual muscles, the multifidus (1.93 ± 1.31%) and tensor fasciae latae (1.92 ± 1.03%) exhibited the highest IMAT percentages.

## Discussion

This study presents comprehensive data from a three-dimensional analysis of thigh muscle volume and intramuscular adipose tissue distribution in a large cohort of 1,287 elderly East Asian hip fracture patients. Overcoming the limitations of conventional two-dimensional cross-sectional analyses, we demonstrate that the complex pattern of muscle atrophy and fatty degeneration observed in high-risk individuals goes beyond simple loss of muscle mass and reflects qualitative deterioration that is heterogeneous across anatomical groups.

Historically, most investigations into sarcopenia and hip fracture risk have relied on single-slice or few-slice cross-sectional area (CSA) measurements, typically at the mid-thigh or L3 vertebral level [23]. While these metrics correlate with total muscle mass to some extent, they fail to capture the heterogeneous distribution of atrophy and fat infiltration that occurs along the longitudinal axis of the femur. Because age-related muscle loss does not occur uniformly, often sparing certain compartments while severely affecting others, extrapolating whole-limb volume from a single 2D plane may introduce significant sampling bias and fail to capture the full heterogeneity of muscle quantity and quality [11]. Furthermore, manual segmentation of the entire thigh is labor-intensive and prone to inter-observer variability, which has previously limited studies to small, healthy volunteer cohorts that do not accurately represent the frail, acutely ill hip fracture population.

Unlike previous studies that extrapolated total volume from selected cross-sectional slices, this study integrated every slice from the iliac wing to below the knee using an AI pipeline to calculate “true 3D volume.” The present analysis showed that the gluteal group had the largest height-normalized muscle volume among the four anatomical groups (855.64 cm³/m²). The large volume of the gluteal musculature, alongside the quadriceps, highlights its potential relevance as a structural imaging marker; however, its relationship with functional recovery requires direct evaluation in longitudinal studies [24].. However, a notable finding of this study was the dissociation between muscle “quantity (volume)” and “quality (IMAT percentage).” The gluteal group, which possessed the largest volume, paradoxically exhibited the highest IMAT percentage (1.43%) [25]. In particular, relatively high IMAT percentages were observed in the multifidus (1.93%) and tensor fasciae latae (1.92%), muscles involved in spinal and pelvic stabilization, compared with primary locomotive muscles such as the vastus medialis (0.38%). This pattern may represent an imaging phenotype relevant to postural function; however, because postural stability, balance, muscle strength, and fall risk were not directly assessed, these findings should be considered hypothesis-generating rather than evidence of a causal mechanism linking muscle composition to fracture vulnerability.

The relatively low absolute IMAT percentages should be interpreted in the context of the operational definition used in this study. Because IMAT was restricted to adipose-range voxels within each individual muscle segmentation mask, while adipose tissue outside the muscle boundary was excluded, these values represent a compartment-specific within-muscle CT measure rather than total thigh adiposity. Accordingly, the absolute magnitude of IMAT percentage may be influenced by the selected attenuation threshold and compartment definition.

Our granular analysis of individual muscles and compartments further elucidates this qualitative-quantitative mismatch (Table 2). Specifically, the gluteal group had the largest height-normalized muscle volume (855.64 ± 216.38 cm³/m²) and also demonstrated the highest IMAT percentage (1.43 ± 0.84%). At the individual muscle level, the gluteus maximus emerged as the largest single muscle (1137.67 ± 379.48 cm³), yet the highest IMAT percentages were observed in the multifidus (1.93 ± 1.31%) and tensor fasciae latae (1.92 ± 1.03%). This pattern of higher IMAT percentages in the multifidus and TFL compared with primary locomotive muscles such as the vastus medialis (0.38 ± 0.32%) suggests heterogeneity in muscle quality across muscle groups with different functional roles. However, because postural stability and muscle strength were not directly assessed, these findings do not establish whether postural stabilization mechanisms are impaired or whether such changes occur earlier than changes in gross motor function.

The observation that more than half of the cohort (52%) maintained pre-injury functional independence (Koval grade 1) despite sustaining a hip fracture highlights the complex relationship between functional status and underlying muscle health. Although many patients were ambulatory before injury, the observed differences in muscle composition suggest that conventional measures of pre-injury functional status may not fully capture variations in muscle quality. Given the cross-sectional design of the present study, the temporal relationship between these imaging findings and functional decline cannot be determined. Furthermore, women in this cohort were significantly older than men despite having similar BMI values, emphasizing the limitations of conventional anthropometric measures in capturing age-related changes in muscle composition. These findings support the potential value of AI-based volumetric muscle profiling for characterizing differences in muscle quantity and composition that may not be adequately reflected by traditional clinical measures alone.

Despite these strengths, several limitations should be acknowledged. First, the cross-sectional nature of this preoperative CT analysis precludes the ability to establish a temporal relationship between muscle loss and the fracture event; it remains unclear whether the observed fatty degeneration preceded the decline in function or was exacerbated by pre-existing comorbidities. Second, while the Cohort is a large-scale registry, the data were collected from tertiary university hospitals, which may introduce a selection bias toward more severe cases or patients with higher surgical candidacy. In addition, a substantial number of initially screened patients were excluded because complete whole-thigh CT imaging was unavailable. Because individual-level data for these excluded patients were not retained in the final analytic dataset, their baseline characteristics could not be formally compared with those of the included cohort. This may have introduced additional selection bias and should be considered when interpreting the generalizability of the findings. Third, CT acquisition and reconstruction parameters were not completely uniform across the multicenter imaging dataset, and formal cross-institutional robustness testing of the AI segmentation model was not performed in the present study. Although the same preprocessing pipeline was applied before segmentation, residual variability related to scanner characteristics and acquisition settings may have affected segmentation performance. Future studies should evaluate segmentation accuracy and failure cases separately across institutions and CT acquisition protocols to further establish the generalizability of the model. Fourth, postoperative mobility, length of stay, rehabilitation outcomes, complications, recurrent falls, and other longitudinal functional outcomes could not be evaluated because these variables were unavailable in the current dataset. Mortality information was available for only a small subset of patients and lacked a sufficiently defined follow-up and censoring framework for reliable outcome analysis. Prospective longitudinal studies are therefore required to determine whether CT-derived muscle measurements predict clinically relevant postoperative outcomes.

## Conclusion

Our findings demonstrate a dissociation between muscle volume and quality, with relatively high fatty infiltration observed in several large or functionally relevant muscle groups, including the gluteal compartment, multifidus, and tensor fasciae latae. These muscle composition patterns may provide additional information beyond muscle volume alone and may help characterize variations in muscle quantity and composition in older adults with hip fracture. However, because postural stability, fall risk, muscle strength, and longitudinal outcomes were not directly assessed, these observations should be regarded as hypothesis-generating and require prospective validation incorporating appropriate control groups and functional outcomes.
